# Learning to Listen: Changes in Children’s Brain Activity Following a Listening Comprehension Intervention

**DOI:** 10.3390/bs14070585

**Published:** 2024-07-10

**Authors:** Michelle Marji, Cody Schwartz, Tri Nguyen, Anne S. Kupfer, Chris Blais, Maria Adelaida Restrepo, Arthur M. Glenberg

**Affiliations:** 1 Department of Psychology, Arizona State University, 950 S. McAllister Ave, Tempe, AZ 85287, USA; michelle.marji@asu.edu (M.M.); codyjackschwartz@gmail.com (C.S.); tri_d_nguyen@brown.edu (T.N.); anne.kupfer@asu.edu (A.S.K.); chris.blais@asu.edu (C.B.); 2 Department of Psychology, University of Wisconsin-Madison, 1202 W Johnson St, Madison, WI 53706, USA; 3 Department of Communication Sciences and Disorders, University of South Florida, 4202 E. Fowler Ave, Tampa, FL 33620, USA; marestrepo@usf.edu; 4 INICO, Facultad de Psicología, Universidad de Salamanca, Avda. de la Merced, 109-131, 37005 Salamanca, Spain

**Keywords:** listening comprehension, simulation, sensorimotor system, effortful control, learning, transfer of training

## Abstract

“Are you LISTENING?” may be one of the most frequent questions preschoolers hear from their parents and teachers, but can children be taught to listen carefully—and thus better comprehend language—and if so, what changes occur in their brains? Twenty-seven four- and five-year-old children were taught a language simulation strategy to use while listening to stories: first, they practiced moving graphics on an iPad to correspond to the story actions, and then they practiced imagining the movements. Compared to a control condition, children in the intervention answered comprehension questions more accurately when imagining moving the graphics and on a measure of transfer using a new story without any instruction and with only immovable graphics. Importantly, for children in the intervention, the change in comprehension from the first to the sixth day was strongly correlated with changes in EEG mu and alpha desynchronization, suggesting changes in motor and visual processing following the intervention. Thus, the data are consistent with our hypothesis that a language simulation listening comprehension intervention can improve children’s listening comprehension by teaching children to align visual and motor processing with language comprehension.

## 1. Introduction

Many children struggle with oral language comprehension, which affects language and reading comprehension abilities later in life [1,2]. Our research investigates whether an embodied language intervention can improve listening comprehension (in contrast to reading comprehension) [3] in typically developing children. Most research investigating neural changes due to interventions involves neural atypical children [4].

Embodied simulation theory proposes that comprehension results from a simulation process: words and phrases drive sensorimotor and emotional cortices into states similar to those when physically sensing and acting in situations [5,6]. Consider the sentence, “You and your partner hold hands while walking on the tropical beach.” The phrase “tropical beach” retrieves modality-specific memories that generate activity in the visual system [7]—this activity is a simulation of the linguistic content. The phrase “while walking” retrieves memories of walking that drive a motor simulation [8,9] and hearing “you and your partner hold hands” invokes an emotional system simulation [10,11]. In this research, we investigate if teaching children to simulate language will result in improved listening comprehension and if this improvement is related to children using motor and visual processing in the brain to simulate language.

In the remainder of this introduction, we first contrast traditional approaches to language comprehension with embodied approaches. Next, we provide an outline of the research, and then we describe the specific research questions examined in this study.

Traditional theories of language comprehension state that humans understand words like a computer processing system using abstract, amodal, arbitrary symbols, as representations of language [12]. According to these traditional theories, sensorimotor brain activity may be associated with language, but it does not create, nor is it necessary for÷ comprehension [12]. One such traditional theory, the latent semantic analysis theory of knowledge, proposes that the meaning of a word is derived from the frequency with which it appears with other words [13]. Thus, the meaning of a word can be determined absent of any examination of the sensation, perception, or possible action connected to the word referents [13]. When seeing the word “happy,” for example, this theory implies that the brain will refer to other abstract, amodal, arbitrary symbols that often appear with the word happy, such as “emotion,” “pleasant,” “good,” etc., to find meaning.

In contrast, the current research is based on simulation theory which states that language understanding occurs by employing perceptual, action, and emotional states that would occur if the scenario were actually happening [5,6]. Although it is reasonable to call this simulation “imagination,” it is important to note that simulation is not always a conscious process.

Simulation theory has been largely supported in past research [8,9]. For example, Glenberg and colleagues [8] tested the prediction that reading sentences describing an act of transfer (physical or conceptual) would generate activity in the motor cortex because the physical transfer of items from one person to another often requires the use of the hands. They found that motor activity in the brain, specifically for hand movement, was greater when reading both concrete and abstract sentences about transfer than sentences without transfer. For example, when considering the sentences “Sally delegated the tasks to Tom” and “Sally gave Tom a pencil,” both the abstract and the concrete sentences lead to comparable motor activity in parts of the brain that activate the hand muscles; however, similar sentences without transfer did not. These results support the claim that abstract sentences are grounded in sensorimotor systems of the brain. In another study, Pulvermüller and colleagues [14] had participants listen to verbs such as “lick, pick, and kick” while brain activity was recorded. Consistent with predictions from simulation theory, tongue, hand, and leg words each activated parts of the brain that corresponded with motor activity during actual tongue, hand, and leg movement, respectively.

It should be noted, however, that some researchers have reported weaker evidence for simulation with abstract sentences compared to concrete sentences (see Willems & Casasanto [15] for one summary of the data). The great majority of the sentences in the children’s stories that we used describe concrete actions and events. Thus, the outcome of this debate does not directly impact the current research.

The Indexical Hypothesis [16] describes the processes by which words are turned into a simulation. First, words are indexed or mapped onto corresponding objects in the world (represented as perceptual symbols in the brain, e.g., Barsalou, [17]). Next, the affordances (or how one can act with those objects) are derived. Finally, the syntax of the sentence (in this case, the who does what to whom) is used to integrate the affordances to create a dynamic simulation. For example, talking to a child about a toy car as they play with it allows the child to map the words “toy car” onto that object [18,19]. If the parent says “Move the toy car to me,” then the child has already (1) indexed the word to the object, (2) understands from experience or knowledge of bodily capabilities that a toy car affords picking up and moving, and (3) comprehends the grammatical structure of the sentence because the child can imagine the integration of actions described to complete the task.

The Indexical Hypothesis and simulation theory are different, in some ways, from traditional theories of discourse comprehension and have some aspects in common. An important similarity is that a simulation is like a discourse model or situation model [20]. Both are meant to represent the meaning of the discourse as derived from the language combined with prior knowledge [21]. A major difference, however, is that traditional theories build representations from abstract symbols, as described above. In contrast, simulation theory builds discourse models from sensorimotor and emotional experiences encoded in modal areas of the cortex. The research reported here tests these accounts of comprehension by including one condition focused on the use of sensorimotor information whereas the other condition is focused on more abstract (in this case, solely verbal) information.

Children often struggle with simulating and indexing while reading [18]. One reason for this struggle is that children often focus on the difficult task of decoding (especially in English with its opaque orthography)—that is, moving from the letters to pronouncing words. Teachers may reinforce decoding both because it is important and because it is an observable behavior that can be reinforced. It is difficult to observe comprehension while it is occurring, thus, when learning to read, children are rarely encouraged to go past pronunciation to meaning. Additionally, the child often has no objects in the environment that correspond to the story, so there are no physical clues about how to index the words. Even when stories have pictures, it is unlikely that the child is always referencing those images for understanding. Note that the problem is not simply that the child does not understand the word meanings, and therefore cannot imagine them. For example, Oakhill and colleagues [22] demonstrate that even when children understand all of the vocabulary in a story, this does not guarantee comprehension.

**Figure 1:**
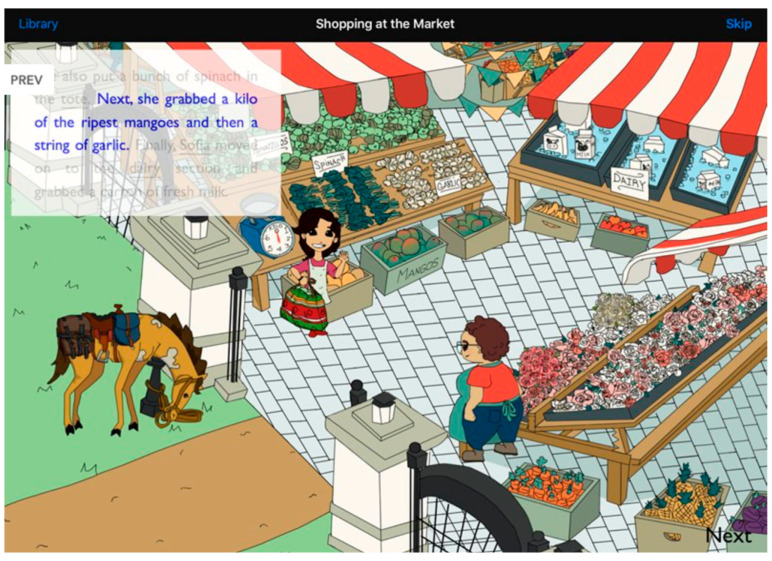
One page from one chapter in the story “A Celebration to Remember.” Children in the intervention condition use physical or imagined manipulation when encountering a sentence in blue font. Here, the child imagines moving the girl to the mangos and then to the garlic. In the control condition, children are instructed to think carefully about the sentences in blue font. Source: EMBRACE iPad application.

Thus, to improve language comprehension, we implemented language simulation training. In our research, children were taught simulation with EMBRACE [18,23], an existing reading/listening comprehension iPad application (app). Here, we briefly describe this simulation intervention as it has been used in past research [18] and the current study. Children in the intervention condition learn to simulate language through physical and imagined manipulations of images using EMBRACE iPad stories. In the physical manipulation (PM) stage, a child listens to texts and has available graphics on the iPad touch screen (see Figure 1). Upon seeing a sentence in a blue font, the child manipulates the graphics to act out the content of the sentence. This manipulation requires children to map nouns to the appropriate graphics (using the visual system of the brain) and to map the syntax of the sentence—the who does what to whom—to their own actions (using the motor system). In the imagined manipulation (IM) stage, the child imagines moving the graphics without touching them, which teaches the child how to internalize the simulation [23,24]. PM and IM improve comprehension, and for good decoders, the skills taught transfer to reading new texts without manipulable graphics [23,24].

Although we frame our work within language comprehension, we believe that it holds important implications for executive function and self-regulation. Self-regulation includes cognitive skills such as planning and organization, directing attention, and impulse control [25]. Learning these skills often involves oral language comprehension, for example, listening to a teacher discuss a plan, or directing attention to a story read by a teacher. Hence, if we can improve listening comprehension, we may indirectly improve self-regulation.

### Research Questions and Hypotheses

In the research reported here, we investigate oral language comprehension with four- and five-year-old children. We studied listening comprehension for several reasons: (a) we know that poor oral comprehension leads to poor reading comprehension [1]. (b) We are interested in an early intervention. Past research suggests that early language development affects later executive functioning (EF), and EF generally develops during preschool and kindergarten [26,27]. (c) We needed a procedure that minimized movements (e.g., eye movements) during EEG measurements that we used to investigate the neural processes changed by the intervention.

Our first research question (RQ1) concerns the efficacy of the embodied intervention. Will it successfully teach children to listen more effectively as measured by post-listening comprehension questions? Simulation theory predicts that children in the intervention condition will show greater comprehension than children in the control condition.

Our second question (RQ2) is whether any improvements from the intervention are correlated with changes in activity in sensorimotor (i.e., visual and motor) systems of the brain as measured using alpha and mu desynchronization. Simulation theory and past research predict a positive correlation between comprehension and desynchronization.

Our third research question (RQ3) asks whether the intervention increases activity in visual and motor systems of the brain compared to the control, or whether the intervention aligns visual and motor activity in the brain with comprehension of the story. The first alternative predicts that after participants complete the intervention, they will show greater visual and motor activity in the brain during a language comprehension task, compared to the control condition where participants would show no change in motor activity over time. The second alternative is that the motor system may be just as active while listening to stories, with or without simulation training. However, simulation training may result in the alignment of motor activity and comprehension as opposed to daydreaming or fidgeting. Simulation theory does not make a clear prediction regarding which of these alternatives is more likely.

## 2. Materials and Methods

To briefly summarize the methods, the children were randomly assigned to the intervention and active control conditions. The measurement of listening comprehension was based on the answers to questions (see Supplemental Materials for the questions) both during the intervention and on a post-intervention transfer test. A covariate Peabody Picture Vocabulary Test performance was used in the analysis of these data. In addition, we used a pre-test/post-test design to measure EEG in dorsal (motor) and occipital (visual) areas while children listened to the texts. Table 1 contains a summary of the day-by-day procedure. All procedures were approved by the Arizona State University Institutional Review Board as STUDY00003921.

### 2.1. Participants

Sample size was based on past research. The use of PM and IM with children often results in large effects compared to a control condition in which children read the text (and re-read critical sentences). For example, Adams, Glenberg, and Restrepo reported a PM partial eta-squared effect size of 0.23 (corresponding to d = 1.09) and a partial eta-squared effect size for IM of 0.25 (d = 1.15) [23]. For the current research, comprehension measures were taken after IM (see below), and so using that estimate of the effect size suggested a sample size of 12 in each group to achieve a power of 0.80. Nonetheless, we elected to modestly increase that sample size given that we had no estimate of the size of the effects for listening comprehension nor for the EEG component of the research. Consequently, prior to the start of data collection, we planned to collect data from 30 children and randomly assign half to each condition. This still-small sample size was chosen due to the complexity of running a 6-day study with children and the limited availability of four- and five-year-old children (and their parents) willing to participate through the Arizona State University Child Labs. We used three waves of data collection in two preschools to reach our goal of 30 participants.

Children were recruited from two preschools from the same institution, the Arizona State University Child Study Lab (CSL) and Child Development lab (CDL), both of which operate during traditional school hours during the spring and fall semesters. In addition, the CDL is open in the summer. Thus, the first wave of data was collected in the spring with children attending the CSL, and second wave in the summer with children from the CDL, and the third wave in the fall with new participants from the CSL. Children from both laboratories were from the same demographic of English as a first language, White or Hispanic children, age 4–5 years (M = 4.72). Of the 30 participants, 18 identified as male and 12 as female, 4 of which came from the CDL and 26 from the CSL. None of the children had known learning disabilities or other conditions that might have affected their participation or learning.

In the first wave of the study, 20 children participated with their parent’s written consent. Children were paired using their ages and their scores on the Peabody Picture Vocabulary Test. In each pair, one child was randomly assigned to participate in the intervention condition, and the other child was assigned to the control condition. Of the 20 children, 13 (8 males and 5 females) completed all six days of the study. 

In the second wave, data were collected from 4 children (4 females) attending the Child Development Laboratory. Children were randomly assigned to the intervention group or the control group. PPVT scores were unavailable for these children. Consequently, data from these children were not used in analyses where PPVT was used as a covariate.

In the third wave of the study, another 16 children agreed to participate in the Child Study Lab. In total, 11 children (9 males and 2 females) completed all six days of the study. As in the first wave, the children were paired using their age and PPVT scores and then randomly assigned to the intervention group or the control group.

If one paired participant did not complete the study, their data were excluded, but their pair was maintained. In the end, 7 participants were unpaired due to 5 dropouts (4 from the intervention condition and 1 from the control condition) and 2 non-viable partner matchings. In total, 16 participants were successfully paired.

Data from one child were removed because that child’s PPVT score, 66, was a low outlier and we were unable to pair the child, resulting in a total of 27 participants (12 in the intervention condition and 15 in the control condition). Outliers were defined (both here and for the EEG data described below) as a score that deviated from the 25th or 75th percentiles by more than 1.5 times the interquartile range. The average PPVT scores of the final participant count were as follows—all participants: M = 103.65, SD = 13.57; control: M = 103.71, SD = 10.47; intervention: M = 103.6, SD = 17.8. We conducted a regression analysis to determine whether PPVT scores were meaningfully different in the intervention and control conditions and found no such effect (*p* = 0.496). All analyses are available on OSF [28].

### 2.2. Conditions

See Table 1 for a summary of the texts used and day-by-day procedures. All children listened to four multi-chapter stories via an iPad program called EMBRACE. The stories were written to be engaging to young children and to emulate children’s literature. They were vetted by parents and teachers in several focus groups. As noted above, children in the intervention condition are taught PM and IM to learn how to both externally and internally simulate. It is possible to manipulate PM and IM independently. In fact, Castro-Alonso, Paas, and Ginns [29] provide an extensive review of how PM-like manipulations alone are educationally effective. Nonetheless, we prefer to have PM and IM in sequential order. First, our initial research [24] showed that PM by itself did not reliably engender transfer (i.e., improve comprehension) for a new text for which the child did not engage in PM. In addition, our observations of young children indicated that they did not understand the IM instruction (“imagine moving the toys”) without previous experience with PM. Finally, we note that Wang, Ginns, and Mockler [30] write that an imagined manipulation (in their work, imagining tracing with the finger) is only effective after a physical manipulation (in their work, using a finger to physically trace part of an image). In fact, in both of their experiments, their imagined procedure always followed their physical manipulation procedure. It is worthwhile to note that, except for a discussion of Montessori’s “sandpaper letters,” all of the work cited by Castro-Alonso et al. [29] and Wang et al. [30] involved research with adults. Thus, our work with preschool children significantly extends the research on manipulation and learning.

Children in our control condition use the iPad to listen to the same stories accompanied by the same graphics. However, when a sentence appeared in blue font, children in the control condition were instructed to pay close attention to the sentence instead of manipulating the graphics. One might question whether this control condition was as engaging as the EMBRACE condition. The story itself was identical (and thus equally engaging) in the two conditions, both texts had the same pictures, and in both conditions, the children worked with the relatively novel iPad. Furthermore, Wall et al. [31] demonstrated that engagement by itself is insufficient to account for differences between conditions that encourage simulation and conditions similar to the control used here. Instead, it was the simulation that produced differences.

### 2.3. Texts and Comprehension Questions

Two of the four stories with similar reading levels, lengths, vocabularies, and genres were used during the EEG sessions on Day 1 and Day 6. These stories were *Bottled Up Joy* (Flesch–Kincaid reading level of 3.6) and *The Lopez Family Mystery* (reading level of 3.1) (see Supplemental Materials for a written transcript of *The Lopez Family Mystery*). We counterbalanced (across children) these narratives for use on Day 1 and Day 6. The first chapter of each of these texts was presented on the iPad, but the remaining chapters of these two texts were presented as an auditory recording during EEG.

Children listened to two other multi-chapter stories on Days 2–5 with the iPad app. That is, the iPad pronounced each sentence and highlighted the pronounced text on the iPad screen. Thus, sentences written in blue font (the cue for children to engage in PM or IM) were readily apparent. Sessions on Days 2–5 were approximately 20 min long, including comprehension questions. Children in the intervention condition engaged in PM for the initial chapters of each story and IM for the remaining chapters. On Days 2 and 3, children listened to a seven-chapter story called *The Best Farm* (Flesch–Kincaid reading level of 2.5) with children in the intervention using PM on Day 2 and IM on Day 3. The second narrative, *Celebration to Remember* (reading level 4.9, Figure 1), had six chapters, and the child listened to three chapters on Day 4 (using PM in the intervention) and Day 5 (using IM in the intervention).

All children answered comprehension questions after listening to (a) chapters used during the EEG sessions, and (b) those chapters for which children in the intervention condition were instructed to use IM. For each chapter, we constructed questions that probed the most important information in the chapter. For each chapter, the questions included at least one that probed verbatim information (i.e., it could have been answered by quoting directly from the story) from a sentence for which children in the intervention did not use PM or IM; at least one that probed verbatim information from a sentence for which children in the intervention did use PM or IM; at least one that probed inferential information (i.e., there was no direct quote that would answer the question) from a sentence for which children in the intervention did not use PM or IM; and at least one that probed inferential information from a sentence for which children in the intervention did use PM or IM (see Supplementary Materials for a complete list of comprehension questions). To maintain adequate power, we collapsed across these distinctions in the analyses. Cronbach’s α for the comprehension questions are in Table 1. This analysis and a factor analysis are available on OSF [28].

### 2.4. Day by Day Procedure

The procedure is summarized in Table 1.

#### 2.4.1. Day 1

A child and parent were escorted to the EEG lab which was decorated with pictures of galaxies, planets, and other space-themed decorations to appear more inviting. Children and parents gave verbal assent and signed consent, respectively. During EEG preparation, the child engaged in a make-believe game we called “Preparing for Space.” The game was used to help children feel comfortable with EEG and to give them something to do during the setup. A cartoon science show was also played in the background for added entertainment during the setup. As the EEG cap was placed on the child’s head, the child was told that it was their make-believe space cap. Electrodes placed behind the ears were noted as creating super ears for space. Gel placed in the electrodes on the scalp was described as space goo, charging up the space cap to be ready for travel. Parents accompanied the children during this process.

To measure motor and visual system activity during comprehension, we examined mu and alpha rhythms with EEG. Mu rhythm is an index of the neuronal activity in the motor cortex [32]: neurons of the motor cortex fire synchronously in the absence of movement, or in the absence of thoughts about movement. Mu power is a measure of this synchronicity [33]. Contrarily, when the motor cortex is active, the neurons begin to fire asynchronously, and mu power decreases, which is mu desynchronization. Previous research has used mu desynchronization to document sensorimotor system activity when reading [34,35]. For example, Moreno and colleagues [34] tested the prediction that mu desynchronization occurs as participants read action-based sentences such as “You will cut the cake.” They found more mu desynchronization during the reading of action-based sentences compared to abstract sentences such as “You will doubt his argument” or sentences about perceptual activities such as “You will notice the bright day” [34]. In line with this past research, we measured mu desynchronization—that is, neural evidence of movement or thoughts about movement—as children listened to stories as our best predictive indicator of whether thoughts about action-based sentences increased after children completed simulation training.

Analogously, we also measured alpha desynchronization, a measure of visual processing [36,37,38]. We reasoned that the EMBRACE intervention teaches children to simulate both movement and visual activity, which should be reflected in mu desynchronization and alpha desynchronization, respectively.

We began the EEG session with a baseline measure of mu desynchronization. The child opened and closed their hand in time with a signal (“an alien hand movement”) to record the baseline measure. Then, the child was asked to look at a visual stimulus (a cross) while remaining completely still. Alpha power (from occipital electrodes) and mu power (from central electrodes), measured while the child looked at the static cross, served as the baseline for computing desynchronization.

Next, children listened to the first chapter of either *Bottled Up Joy* or *The Lopez Family Mystery* (counterbalanced) using the iPad, without EEG recording and answered comprehension questions. The first chapter was used to teach the children how to use the iPad and to orient them to the story. Children were taught to press the “next” button at the bottom of the screen at the end of each sentence (when the iPad stopped playing the sentence). Some sentences appeared in black font and other sentences were written in blue. All children were told to think about blue sentences carefully.

After the first chapter and before the second chapter, we began EEG recording. So as not to interfere with EEG data collection, children listened to the rest of the recorded story sentence-by-sentence without the iPad. They did not engage in PM or IM. At least three chapters were used; additional chapters were used depending on the child’s tolerance (up to 6 chapters total). We aligned event-related potentials with all of the verbs in the story.

After listening to each chapter, the children were asked comprehension questions and given a sticker. For each comprehension question, children were first asked to provide a free-response answer, and each correct answer received two points. If the child answered incorrectly or was unable to provide a response, the child was given a forced-choice option between two answers. A correct forced-choice response received one point and an incorrect response received zero points. The total points for the day were divided by the number of points possible for that day to calculate the child’s comprehension score. In total, children were asked between 38 and 42 questions on each of Days 1 and 6 depending on the child and parent’s desire to continue. One participant needed to leave their session early on Day 6; thus, they did not complete 16 questions out of 42. All other children completed all comprehension questions on Days 1 and 6. Children were given a large sticker at the end of the session.

#### 2.4.2. Days 2–5

The intervention group received four days of simulation training with the iPad app EMBRACE. During PM, children manipulated the graphics on the iPad screen using their fingers. The control group also engaged in the training for four days, but without PM or IM.

On Days 3 and 5 (the second day of listening to chapters from *Best Farm* and *Celebration to Remember*, respectively), children in the intervention condition were instructed to use imagined manipulation (IM). That is, when seeing a sentence in blue font (while listening to the sentence), they were asked to imagine how they would move the pictures, but they were unable to physically move them. In each of the IM chapters, children in the intervention condition saw, twice per chapter, a pop-up question asking them what they imagined. Children chose between two images but were not told if their choice was correct. This feature is built into the EMBRACE program but was not used in data analyses. (see Supplemental Information).

When children in the control condition saw a blue sentence, they were instructed to think about it carefully, but they were given no instruction to use PM or IM. Otherwise, children in the control condition were treated identically to children in the intervention condition. All children answered 13 comprehension questions on Day 3 and 14 questions on Day 5.

#### 2.4.3. Day 6

The procedure was identical to that of Day 1 but with two exceptions. First, children listened to a new story, either *Bottled Up Joy* or *Lopez Family Mystery* (counterbalanced with the narrative used on Day 1). As on Day 1, children were not given any instruction to use PM or IM on Day 6. Second, before leaving, the children were rewarded with a space book for completing the experiment. See Table 2 for data on the time interval between days of the experiment. (We conducted a linear regression to test whether the duration between days was significantly different between the control and intervention groups. We found no significant difference for all days [28]).

### 2.5. Pre-Analysis Data Preparation

Analyses of the comprehension data were conducted after summing the raw comprehension scores for each child on each of Days 1, 3, 5, and 6 (those days on which there was no PM), and then we computed an average percentage correct for each day. In addition, a change score was computed as the Day 6 percentage minus the Day 1 percentage. Thus, a positive change score reflects an increase in correct comprehension question answering.

Nine electrodes from a child-sized 32-channel 10-20 cap were gelled and measured for impedances, including the central electrodes C3, CZ, and C4, which measure activity over the left, middle, and right motor cortex, respectively, the occipital electrodes O1, OZ, and O2, which measure activity over the left, middle, and right occipital cortex, respectively, and the ground. Additionally, two electrodes were placed on the left and right mastoid processes, M1 and M2, respectively, as a reference signal. This method was chosen due to funding constraints and child comfortability. We aimed to keep the child’s attention for as long as possible without losing attention during a prolonged EEG setup. The EEG data were recorded at 1000 Hz and bandpass filtered between DC and 400 Hz in reference to M1. Offline, the data were bandpass filtered between 0.1 and 30 Hz and re-referenced to the average of M1 and M2. Because the participants were four- and five-year-old children, facial electrodes were not used, so blinks and eye movement artifacts were not removed. Although this may be deemed problematic, prior to data collection, we examined the impact of both artifacts in the 8–13 Hz range of interest and found that they were minimal and that most of the activity was <3 Hz. We attempted to keep all impedances below 10 kΩ if the child and parent were willing. Continuous analog-to-digital conversion of the EEG and stimulus trigger codes was performed online by the Neuroscan acquisition interface system. EEG data were transformed with a Fast Fourier Transform which extracted the power of the alpha frequency range.

The data were decomposed into their time-frequency representation via wavelet convolution [39]. Specifically, the power spectrum of the EEG signal was multiplied by the power spectrum of the set of complex Morlet wavelets [ei2πtf e − t2/(2σ2), where t is time, f is frequency, which increased from 2 to 40 Hz in 40 logarithmically spaced steps, and σ defines the width of each frequency band, set according to n/(2πf), where n is the number of wavelet cycles, and increased from 4 to 10 in logarithmic steps], and the inverse fast Fourier transform was then taken. From the resulting complex signal, an estimate of frequency-band-specific power at each time point was defined as the squared magnitude of the result of the convolution [real[z(t)]2 + imaginary[z(t)]2]. Only activity corresponding to the frequency band of interest for each child (see below) was examined. All power values in the time-frequency representation were normalized to the average pre-stimulus baseline power at each frequency band.

Triggers were set at the onset of the presentation of the main verb in each sentence, and data were separately collated for active and non-active verbs (117 total; 48 active, 69 non-active) for exploratory analyses [14]. More specifically, we distinguish between active verbs such as “run,” “jump,” and “kick,” and nonactive verbs such as “want,” “need,” and “have.” This is to determine if there was a significant change in motor system activity for active verbs before and after the EMBRACE intervention and if this change would or would not occur with non-active verbs. There were no significant differences in mu power before and after the intervention for active and non-active verbs.

To effectively measure the mu rhythm in children, we followed past research. In adults, mu rhythm was found at a frequency of 8–13 Hz over the somatosensory cortex [40]. For children, however, the mu frequency band tends to be in a lower frequency range [34]. In a meta-analysis of mu rhythm bands of children from infancy to 4 years of age, it was found that 4-year-old children express a mu rhythm at 6–10 Hz with a peak of 9 Hz [41]. Thus, we examined the mu rhythm in the 6–10 Hz frequency band. For each child, we visually determined the frequency band to the closest whole number (e.g., 9 Hz) within the 6–10 Hz range that showed the strongest signal during viewing of the static noise stimulus on Day 1.

For both mu and alpha, we restricted measurement to the interval between 200 and 500 ms following the onset of the verb [14].

The EEG data were subjected to a series of steps before testing the research questions. First, because of excessive movement over the course of the session, all children showed evidence of “dropped” electrodes as indicated by very large positive and negative voltages. Consequently, the distribution for each electrode on each day, for each stimulus trial, and for each participant was examined for outliers (a score that deviated from the 25th or 75th percentiles by more than 1.5 times the interquartile range). The 125 outliers (out of a total of 1296 observations) were removed. Second, as recommended for the analysis of data sets with missing data and as carried out in previous research [42,43] these missing data were then replaced using a multiple imputation algorithm (SPSS v 25) using the remaining EEG data, as well as the child’s condition, age, PPVT, gender, duration in the experiment, and comprehension scores. Third, a proportion-alpha variable was computed by dividing a target value (e.g., Day 1, O1 electrode, action verb) by the corresponding value in the static measurement (e.g., Day 1, O1 electrode, static). Fourth, the proportion-alpha variables were subjected to four factor analyses: one for the occipital electrodes on Day 1, one for the occipital electrodes on Day 6, one for the central electrodes on Day 1, and one for the central electrodes on Day 6. The first factor score (which had an eigenvector > 1 in all of the analyses) in each of these factor analyses was used in further analyses. Fifth, we computed change scores to index change in desynchronization from Day 1 to Day 6. The change in the occipital desynchronization is the occipital factor score on Day 1 minus the occipital factor score on Day 6. Similarly, the change in the motor desynchronization is the central factor score on Day 1 minus the central factor score on Day 6. Given the direction of this subtraction, if there is greater alpha and mu desynchronization (a smaller proportion) on Day 6 than on Day 1, then the change score is positive. The correlation data reported later use these change scores. However, the factor analysis forces scores to have a mean of zero, and thus these scores are inappropriate for testing the third research question of whether there is an increase in sensorimotor activity from Day 1 to Day 6. Thus, the proportion-alpha values (from the third step) were used in the analysis directed at this question.

## 3. Results

**Figure 2:**
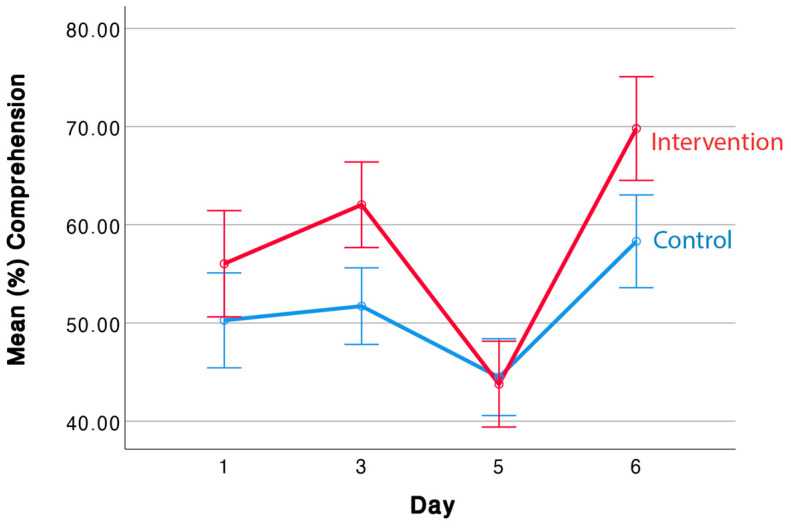
Performance on comprehension questions. Error bars are ± one standard error.

Figure 2 presents data relevant to the first research question: will the embodied intervention successfully teach children to listen more effectively as measured by post-listening comprehension questions? On Day 1, children listen to stories before any instruction on manipulation. Hence, any difference between the intervention and control conditions is due to random variability, and in fact, the difference between the conditions is not statistically significant, F(1, 25) = 0.63, *p* = 0.43. The reliability of the measurement (see Table 1) is adequate; hence, the null effect is not due to low reliability. Because of the small advantage of children in the intervention, we decided to use performance on Day 1 and the children’s scores on the Peabody Picture Vocabulary Test (PPVT) as covariates in further analyses. 

Children in the intervention condition received training in PM and IM on Days 2–5, but their comprehension was only tested when they engaged in IM on Days 3, 5, and 6. In the control condition, children listened to the same stories and saw the same graphics, but there was no instruction regarding manipulation.

No outliers were detected in comprehension question scores for any participants. Performance on Day 5 is very poor and equivalent across the two conditions. In retrospect, the reason is clear. This text had a Flesch–Kincaid reading level (grade 4.9, approximately 10 years old) well beyond the age of the preschool children. In addition, it is written to evoke Mexican culture and contains many vocabulary words (e.g., “mole,” “champurada,” “peso,” and “kilo”) that were unfamiliar to the predominantly White children in the university’s Child Study Lab and Child Development Lab (see Figure 1 for a screenshot taken from this story). Although we expect simulation to help with both easy and (somewhat) difficult texts, it cannot help if children cannot engage in simulation. When faced with words such as “champurada” and “peso,” which name concepts for which the children have had no experience, then it is next to impossible to simulate. Probably for these reasons, the data show low reliability (see Table 1).

We analyze the data from Days 3 and 6 (for which the comprehension measures were adequately reliable, see Table 1) using an analysis of covariance, with Day 1 recall and PPVT as covariates, and Day (3 or 6) as a within-subject variable and condition (control or intervention) as a between-subjects variable. There is a significant effect of condition, *F*(1, 19) = 6.28, *p* = 0.022, *d* = 1.05. As is apparent in Figure 2, performance is higher in the intervention condition. No other effects or interactions are significant. Importantly, the benefit of the intervention is also significant on Day 6 alone, *F*(1, 20) = 5.33, *p* = 0.03, *d* = 1.09. On this day, children listen to a new story without any instruction regarding physical or imagined manipulation; thus, Day 6’s performance shows a transfer of training. 

**Figure 3:**
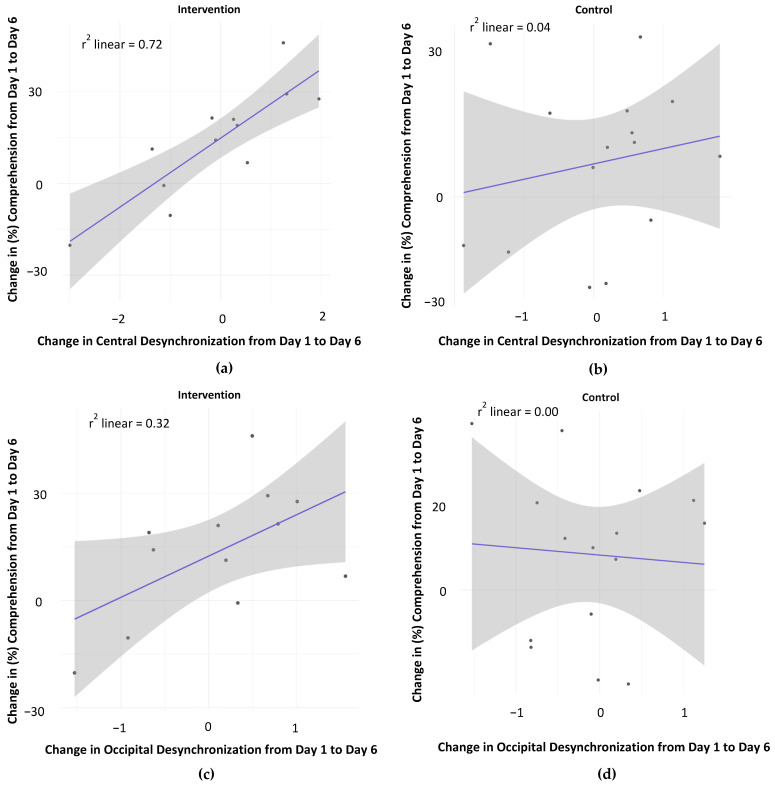
(**a**) Relation between change in text comprehension and change in mu desynchronization measured at the central electrodes for the intervention condition; (**b**) relation between change in text comprehension and change in mu desynchronization measured at the central electrodes for the control condition; (**c**) relation between change in text comprehension and change in alpha desynchronization measured at the occipital electrodes for the intervention condition; (**d**) relation between change in text comprehension and change in alpha desynchronization measured at the occipital electrodes for the control condition. In each panel, the shaded region corresponds to the 95% confidence interval for the slope of the regression line.

Figure 3a–d present data relevant to the second question: are improvements in comprehension following the intervention correlated with changes in activity in the sensorimotor cortex? Desynchronization (i.e., reduction in power) of mu and alpha rhythms are generally interpreted as reflecting, respectively, active motor and visual processing [34,44,45]. For each child, we compute the change in desynchronization from Day 1 to Day 6 as well as the change in comprehension performance from Day 1 to Day 6, and then we examine the relation between these two change scores.

According to simulation theory, the comprehension of action-based language requires simulation using the motor system. Thus, we predict that improvements in comprehension would be accompanied by changes in motor system activity: there should be a positive correlation between change in comprehension and change in mu desynchronization measured at the central electrodes (presumed to index motor system activity). This prediction is robustly supported by the data in Figure 3a. That is, for the intervention condition, the linear relation was statistically significant, *r*(11) = 0.85, *p* < 0.001; *r_s_*(11) = 0.78, *p* = 0.003 (*r* is the Pearson correlation and *r_s_* is the Spearman correlation). In contrast, as illustrated in Figure 3b, there is no significant linear relation in the control condition, *r*(14) = 0.19, *p* = 0.49; *r_s_*(14) = 0.30, *p* = 0.28. Furthermore, the positive correlation between change in comprehension and change in mu desynchronization is greater in the intervention condition compared to the control condition, *z* = 2.29, *p* = 0.01.

For language that is highly imageable (such as that used in these texts), the simulation will engage perceptual systems. Thus, we predict that there will also be a positive correlation between the change in comprehension and the change in alpha desynchronization measured at the occipital electrodes (presumed to index visual system activity). As shown in Figure 3c, this prediction is supported but to a lesser extent than in the motor cortex, *r*(11) = 0.57, *p* = 0.05; *r_s_*(11) = 0.54, *p* = 0.07. Again, however, as illustrated in Figure 3d, there is no significant linear relation in the control condition, *r*(14) =−0.07, *p* = 0.82; *r_s_*(14) = 0.00, *p* = 0.99. The positive correlation between change in comprehension and change in alpha desynchronization is greater in the intervention condition, compared to the control, but this difference does not reach statistical significance, *z* = 1.54, *p* = 0.06.

The third question is whether the relations evident in Figure 3a–d reflect increased activity in the sensorimotor cortex from Day 1 to Day 6 or better alignment of cortical activity with the goal of comprehension. To discriminate between these two hypotheses, we conduct, for the intervention condition, an analysis of variance on the proportion power compared to baseline (step 3 in the EEG analyses described above). The analysis includes four within-subject factors, Day 1 versus Day 6, occipital versus central electrodes, left versus middle versus right electrode location, and action verb versus non-action verb. There are no significant main effects or interactions, that is, we do not find evidence for an increase in desynchronization. In contrast, the data in Figure 3a–d are consistent with the alignment hypothesis: the intervention teaches children how to simulate by using visual and motor areas of the brain in the service of comprehension, rather than simply increasing visual and motor cortex activity. In other words, the data support the claim that the EMBRACE intervention teaches children to use the visual cortex to imagine the visual content of the language and to use the motor cortex to imagine the actions suggested by the text.

## 4. Discussion

This research resulted in two main discoveries. First, compared to children in the control condition, children in the simulation intervention answered comprehension questions for age-appropriate texts with significantly greater accuracy (Figure 2). Second, this enhanced listening comprehension was associated with changes in EEG activity that indexes sensorimotor activity, that is, children who answered comprehension questions more accurately showed greater alignment with sensorimotor activity (Figure 3). We discuss the novelty of these findings, their theoretical implications, and the limitations of the research.

Most of the research conducted with young children and language comprehension has been in the context of reading comprehension. Nonetheless, as the results in Figure 2 illustrate, listening comprehension is mutable: it may be improved with an appropriate intervention.

Interventions that include measures of neurophysiological change are rare, and virtually all of that research has been conducted with children with atypical development. As one example, Romeo and colleagues [46] studied the effects of a reading intervention for children with reading disabilities and reported effects on cortical thickness. As another example, Leppänen [47] reviewed six articles in a Special Issue on brain-event-related potentials as biomarkers of language and literacy development, feedback, and intervention. Of the six studies, four involved atypically developing children including specific language impairment, reading difficulties, and Williams Syndrome. None of the studies examined discourse understanding. Thus, our results may be one of the first to demonstrate an embodied intervention resulting in improved listening comprehension for typically developing children and to align that improvement with measures of brain activity.

The present study tested several predictions from the theory of embodied cognition and simulation as applied to language. First, the theory predicts that teaching simulation will improve language comprehension. The data in Figure 2 support this prediction, at least for age-appropriate texts. Second, the theory predicts that improvements in comprehension will be accompanied by changes in the sensorimotor cortex. The data in Figure 3 are consistent with this prediction.

In contrast, the results are less consistent with more traditional approaches to language comprehension based on abstract symbols and distributional semantics [12,13]. On these accounts, sensorimotor activity is unrelated to language comprehension. Although these accounts might explain the improved comprehension (Figure 2) due to enhanced attention to the texts, these accounts would not predict a correlation between the improved comprehension and activity in sensorimotor systems (Figure 3).

As we noted above, the results for Day 6 (Figure 2) indicate a type of transfer. That is, children in the intervention condition outperformed children in the control condition even though none of the children were given special instructions on Day 6 and none of the children engaged in explicit PM. Furthermore, the Day 6 story was unrelated to stories used on other days. So, what could have transferred? We infer that in the intervention condition, children learned new strategies for listening and comprehending. That is, they learned to use sensorimotor systems to engage in simulation while listening. The EEG data reported in Figure 3 are strongly consistent with this explanation. Once this strategy is learned, it appears that children may transfer this learning to the understanding and simulation of new stories.

Several limitations should be addressed in future research. Our sample size was small, and thus statistical power was low. We did take some precautions to increase power. For example, we paired students by PPVT scores. Unfortunately, due to a variety of issues (e.g., not all participants had PPVT scores), 11 of the 27 children were not paired in the final analyses. We also used age to pair children. While valuable, age is not as useful as considering time in the preschool program. Also, participants in this research were mostly White or Hispanic. Children fidgeted during data collection leading to noisy EEG data. Importantly, because our children had limited tolerance for the EEG equipment, we were unable to consistently gel all electrodes which precluded sophisticated source localization. Children also completed the 6-day study over different lengths of time ranging from 7 to 110 days. (110 is a clear outlier (the next highest duration was 48 days). All analyses were reconducted with this outlier excluded and with duration in the study included as a covariate. There were only minor changes in the significance levels, none of which affected the conclusions we have drawn. The re-analyses are available at [28]). This may have affected the quality of the simulation training and how much information children retained from that training. It is also important to note that more physical engagement in the stories may have contributed to more engagement in the activity overall. Children in the intervention moved images on the iPad, often repeating the movement until the correct manipulation was complete, whereas children in the control were instructed to think about specific sentences carefully and press the “Next” button to move to the next page of the stories. One may argue that children in the intervention condition were more engaged with the stories or were instructed to identify characters and objects in the story to complete the physical manipulation task. Importantly, however, none of the children interacted with the iPad during EEG measures on Days 1 and 6, and protocols were the same for both groups on these days. Furthermore, as demonstrated by Wall et al. [31], engagement alone cannot explain the effects of simulation. Finally, we note that the intervention was relatively brief (four sessions), and we did not measure any long-term effects of the intervention.

Learning to listen is related to self-regulation and effortful control which develop in the first years, particularly in preschool and kindergarten [26] and continue developing into adolescence [48] and probably adulthood [49]. In turn, effortful control is correlated with social competence, social cognition, maladjustment, and school outcomes [50]. Our results suggest that teaching embodied simulation strategies is almost certainly a more effective method for enhancing effortful control than a hectoring “Are you LISTENING?”.

## 5. Conclusions

We draw several conclusions from this research. First, an embodied listening comprehension intervention improves children’s comprehension of spoken narratives. Second, that improvement is accompanied by alignment of activity in motor and visual processing, suggesting that language comprehension is based on sensorimotor processing [51]. 

## Figures and Tables

**Table 1:** Six-day experimental procedure for the intervention condition and the control condition.

Day (Reliability)	Text	Intervention Condition Instruction	Control Condition Instruction
**Phase 1: EEG Baseline Measure**			
1 (0.83)	Bottled Joy or Lopez	Think carefully about blue sentences	
**Phase 2: Intervention and Control**			
2	Best Farm	PM	Think carefully about blue sentences
3 (−0.01)	Best Farm	PM and IM	Think carefully about blue sentences
4	Celebration	PM	Think carefully about blue sentences
5 (0.54)	Celebration	PM and IM	Think carefully about blue sentences
**Phase 3: EEG Post Measure**			
6 (0.88)	Lopez or Bottled Joy	Think carefully about blue sentences	

Note: On Days 1, 3, 5, and 6, children answer comprehension questions. Reliability is Cronbach’s α. The negative reliability for Day 3 indicates that the model assumptions were violated. Bottled Joy = *Bottled Up Joy*; Lopez = *Lopez Family Mystery*; Celebration = *A Celebration to Remember*; PM = physical manipulation; IM = imagined manipulation.

**Table 2:** Duration of time in days between each day of the study.

	Day 1–2	Day 2–3	Day 3–4	Day 4–5	Day 5–6
**Average time interval**	5.33	3.96	5.44	4.59	6.81
**Min**	1	1	0	1	1
**Max**	27	25	70	27	23

Note: All analyses were reconducted with the participant with the longest duration (110 days) excluded and with duration in the study included as a covariate. There were only minor changes in the significance levels, none of which affected the conclusions we have drawn.

## Data Availability

All data are available at the Open Science Framework [28].

## Supplementary Materials

The following supporting information can be downloaded at: https://www.mdpi.com/article/10.3390/bs14070585/s1, Table S1: The Lopez Family Mystery Used on Day 1 or Day 6; Table S2: Comprehension Questions Used on Day 1 and Day 6.
