# Supplementary material for: Learning to Listen: Changes in Children’s Brain Activity Following a Listening Comprehension Intervention
Source: Behav Sci (Basel). 2024 Jul 10;14(7):585. doi: 10.3390/bs14070585 (PMC11273652; doi:10.3390/bs14070585)
Supplement: Supplementary file 1 [file behavsci-14-00585-s001.zip › behavsci-2957471-supplementary.pdf]

Table S1: The Lopez Family Mystery Used on Day 1 or Day 6

Chapter 1: The Lopez Family

The Lopez family lives in a nice house.
Martin is the father, and he is a policeman. He works every day.
In the morning, Martin leaves for work. He takes his keys from the hook in the kitchen and gets into his car.
He drives to the front of the house and waves goodbye to the family from the car.

- IM-check: Martin next to car vs Martin in car*

Rosa, the mother, comes out to say goodbye.
Rosa is a lawyer, but she works from home to take care of the baby.
The Lopez family has two pets.
The bunny, whose name is Lola, jumps up to visit the baby.
Paco the dog runs outside to lick Martin goodbye.

- IM-Check: Paco and Martin vs Lola and Martin*

Soon, the whole Lopez family will have a mystery to solve!

Chapter 2: Missing Keys

The next day, Martin woke up and washed his face in the bathroom.
Then, he brought the baby down to the kitchen for breakfast and put her into the highchair.

- IM-check: baby in highchair vs baby next to highchair*

After breakfast, Martin walked over to get his keys from the hook.
But, the keys were gone!
Martin looked in the living room for the keys.
Paco sniffed for the keys outside.

- IM-check: Paco near tree vs Lola near tree*

Lola the bunny looked for the keys on the top floor where the baby sometimes plays.
Where were the missing keys?

Chapter 3: Is Paco a Thief?

Paco smelled something. Maybe it was the keys.
He ran to the tree and sniffed some more. (M)

Then he saw the keys in the tree!
He barked and barked, and suddenly, the keys dropped onto the ground.

- IM-check: Keys on hook vs keys on ground near tree*
- *Animate keys falling*

Paco picked up the keys and ran toward the house.

- IM-check: Paco facing house vs Paco facing away from house*

Martin heard the barking and went outside.
Martin said, “Paco, you are a bad dog for stealing my keys! You will have to stay outside all day.” Paco was very sad.

Chapter 4: More is Missing!

The next morning, Rosa got up early and went to the kitchen.
She was going to make pancakes for the whole family. She walked to the sink to get her silver spoon for stirring.
But the spoon was missing!
Rosa went to the living room and said, “Paco, did you take the silver spoon?”

- IM-check: Rosa and Paco facing each other vs Rosa and Lola facing

each other*
Paco ran out to the tree and barked.
He barked and barked, and the silver spoon dropped to the ground!
Animate spoon falling
Paco picked up the spoon and carried it to Rosa.

- IM-check: Paco & spoon (on right) facing Rosa vs Paco & spoon

facing away from Rosa*
Rosa said, “Paco, you are a bad dog for stealing my silver spoon. You will have to stay outside all day.”

Chapter 5: The Baby's Rattle is Gone, Too!

Later in the day, the baby wanted to play with her rattle.
She looked in her crib, but it wasn’t there.
She looked in the living room, but she couldn’t find her rattle there either. The baby started to cry.
Lola the bunny jumped into the baby’s lap to comfort her.

- IM-check: Lola in baby's lap vs Lola on baby's head*

Rosa went into the living room when she heard the baby cry.

Paco heard the baby cry, too.

He looked up in the tree and there was the baby's rattle!

- IM-check: Paco and tree vs Lola and tree*

Paco barked and barked, and the rattle dropped to the ground.

- *Animate rattle falling*

Rosa picked up the baby and went out to see why Paco was barking.

Lola went out, too.

Chapter 6: The Mystery is Solved

Martin drove home and put his keys on the hook.

- IM-check: keys on hook vs keys next to hook*

Paco, Rosa, Lola and the baby were by the tree, and Martin went out to join them.

Just then, a bird flew out of the tree and into the kitchen.

The bird took the keys and flew to its nest.

- IM-check: bird and keys vs bird and spoon*

Paco barked and barked, and the bird dropped the keys to the ground.

- *Animate keys falling*

Now everyone understood that the bird loved to take shiny things, and the bird was the thief!

So, the mystery was finally solved. And, Paco was a hero!

That night, Paco got a big steak dinner for a reward.

*Lines with asterisks refer to the two images that pop up on the iPad after imagined manipulation. Children are directed to choose the image that corresponds to what they imagined.

Table S2: Comprehension Questions Used on Day 1 and Day 6

The Lopez Family Mystery

Chapter 1 The Lopez Family

1) NV. How many pets does the Lopez family have?
- Child Free Response ________
- Forced Choice Answer:
*A: two pets

B: one pet

2) AV. What does Martin grab from the kitchen?
- Child Free Response ________
- Forced Choice Answer:
*A: his keys
B: the baby

3) AV. Who visits the baby?
- Child Free Response ________
- Forced Choice Answer:
*A: Lola, the bunny
B: Paco, the dog

4) AV. Where does Martin drive his car?
- Child Free Response ________
- Forced Choice Answer:
*A: to the front of the house
B: to the store

5) NI. What will the family be doing soon?
- Child Free Response ________
- Forced Choice Answer:
*A: solving a mystery
B: going to the park

6) AI. Why does Martin grab his keys?
- Child Free Response ________
- Forced Choice Answer:
*A: to drive his car
B: to open the front door

7) NV. Why does Rosa work from home?
- Child Free Response ________
- Forced Choice Answer:
*A: to take care of the baby
B: to take care of Paco

<u>Chapter 2 Missing keys</u>

1) AV. Where did Martin bring the baby?
- Child Free Response ________
- Forced Choice Answer:
*A: to the highchair in the kitchen

B: to the couch in the living room

2) NV. Where does the baby sometimes play?
- Child Free Response ________
- Forced Choice Answer:
*A: on the top floor
B: outside

3) AV. Who looked for the keys on the top floor?
- Child Free Response ________
- Forced Choice Answer:
*A: Lola, the bunny
B: Paco, the dog

4) AV. Why did Martin go to the living room?
- Child Free Response ________
- Forced Choice Answer:
*A: to look for the keys
B: to look for Lola

5) AV. Why did Martin put the baby in the highchair?
- Child Free Response ________
- Forced Choice Answer:
*A: so she could eat breakfast
B: so she could eat dinner

6) AI. How did Paco try to help Martin?
- Child Free Response ________
- Forced Choice Answer:
*A: Paco sniffed for the keys outside
B: Paco looked for the keys on the top floor

<u>Chapter 3 Is Paco a Thief?</u>

1) NV. What did Paco smell?
- Child Free Response ________
- Forced Choice Answer:
*A: keys
B: food

2) AV. Why did Martin go outside?
- Child Free Response ________
- Forced Choice Answer:
*A: because he heard barking

B: to get in his car

3) NV. Why did Martin think Paco was a bad dog?
- Child Free Response ________
- Forced Choice Answer:
*A: for stealing the keys
B: for barking

4) NV. How did Paco feel at the end of this chapter?
- Child Free Response ________
- Forced Choice Answer:
*A: sad
B: happy

5) NI. How did Martin punish Paco?
- Child Free Response ________
- Forced Choice Answer:
*A: by making him stay outside all day
B: by making him stay inside all day

6) NV. Where did Paco find the keys?
- Child Free Response ________
- Forced Choice Answer:
*A: in the tree
B: on the top floor

7) AV. What did Paco do when he saw the keys?
- Child Free Response ________
- Forced Choice Answer:
*A: barked and barked
B: jumped up and down

8) AI. Why did Paco run to the tree?
- Child Free Response ________
- Forced Choice Answer:
*A: he smelled the keys in the tree
B: he liked to bark at the tree

<u>Chapter 4 More is Missing!</u>

1) NV. What time of day was it?
- Child Free Response ________
- Forced Choice Answer:
*A: morning

B: afternoon

2) NI What did Rosa think her family wanted in the morning?
- Child Free Response ________
- Forced Choice Answer:
*A: breakfast
B: to play

3) NV. What did Rosa want to use the spoon for?
- Child Free Response ________
- Forced Choice Answer:
*A: stirring
B: scooping

4) NV. What was missing from the sink?
- Child Free Response ________
- Forced Choice Answer:
*A: a spoon
B: a fork

5) AI. Why did Rosa go into the living room?
- Child Free Response ________
- Forced Choice Answer:
*A: to talk to Paco the dog
B: to look for the spoon

6) AV. Who found the spoon?
- Child Free Response ________
- Forced Choice Answer:
*A: Paco
B: Rosa

7) AV. What happened to the spoon when Paco barked?
- Child Free Response ________
- Forced Choice Answer:
*A: it dropped to the ground
B: it got stuck in the tree

8) NV. Who did Rosa blame for stealing the spoon?
- Child Free Response ________
- Forced Choice Answer:
*A: Paco
B: Lola

Chapter 5 The Baby's Rattle is Gone, Too!

1) AV. Who looked in the living room for the rattle?
- Child Free Response ________
- Forced Choice Answer:
*A: the baby
B: Lola, the bunny

2) AV. Why did Lola jump in the baby's lap?
- Child Free Response ________
- Forced Choice Answer:
*A: to comfort the baby
B: because Lola was scared

3) NI. Why was the baby sad?
- Child Free Response ________
- Forced Choice Answer:
*A: she couldn't find her rattle
B: she was hurt

4) NV. What did Paco hear?
- Child Free Response ________
- Forced Choice Answer:
*A: the baby cry
B: the baby giggle

5) NV. What did Rosa hear outside?
- Child Free Response ________
- Forced Choice Answer:
*A: Paco barking
B: a bird chirping

6) AI. How did the baby get outside?
- Child Free Response ________
- Forced Choice Answer:
*A: Rosa carried her
B: she crawled out

Chapter 6 The Mystery is Solved

1) NI. Who stole the rattle?
- Child Free Response ________
- Forced Choice Answer:
*A: the bird

B: Paco

2) NI. Why was Paco a hero?
- Child Free Response ________
- Forced Choice Answer:
*A: for helping the family solve the mystery
B: for returning the things that he stole

3) NI. What was the mystery?
- Child Free Response ________
- Forced Choice Answer:
*A: finding out who stole the shiny things
B: finding out why the baby was crying

4) NV. How was Paco rewarded?
- Child Free Response ________
- Forced Choice Answer:
*A: with a big steak dinner
B: with a chew toy

5) AV. Who put the keys on the hook?
- Child Free Response ________
- Forced Choice Answer:
*A: Martin
B: Rosa

6) AI Why did the bird fly into the kitchen?
- Child Free Response ________
- Forced Choice Answer:
*A: to get the keys
B: to get some food

7) NV. What did the bird like about the things it took?
- Child Free Response ________
- Forced Choice Answer:
*A: they were shiny
B: they were small

Bottled up Joy

<u>Chapter 1: The Lucky Stone</u>

1. (NI) What can Lucas see from the hill?

- Child Free Response ________
- Forced Choice Answer:
*A) A river
B) A bottle

2. (AV) Why does Lucas walk down the hill most afternoons?
- Child Free Response ________
- Forced Choice Answer:
A) To grab lunch
*B) To watch the sunset

3. (NV) What was so special about this afternoon?
- Child Free Response ________
- Forced Choice Answer:
*A) Lucas found a bottle floating in the water
B) Papa was home

4. (AV) Where did Lucas bring the bottle?
- Child Free Response ________
- Forced Choice Answer:
A) Up to his room
*B) Home to his papa

5. (AV) What did Papa do with the bottle?
- Child Free Response ________
- Forced Choice Answer:
*A) He opened it
B) He drank it

6. (AV) What tumbled across the floor?
- Child Free Response ________
- Forced Choice Answer:
*A) A note and stone
B) A bottle

7. (AV) What did Papa pick up from the ground?
- Child Free Response ________
- Forced Choice Answer:
*A) The note
B) The stone

8. (From 2 sentences A&N, I) Why did Lucas think he would be granted three wishes?
- Child Free Response ________
- Forced Choice Answer:

*A) Because he found the lucky stone
B) Because Papa gave him the lucky stone

Chapter 2: Magic Toys

1. (NI) Why did Lucas go back to the river?
- Child Free Response ________
- Forced Choice Answer:
A. To return the bottle
*B. To think about his wishes

2. (NV) What did Lucas wish for?
- Child Free Response ________
- Forced Choice Answer:
*A. A room full of the best toys
B. A bigger ball

3. (NV) How did Lucas feel about the toys when they appeared?
- Child Free Response ________
- Forced Choice Answer:
*A. He couldn't wait to start playing with them
B. He wasn't happy with them

4. (AV) Which toy did Lucas try to play with first?
- Child Free Response ________
- Forced Choice Answer:
*A. The ball
B. The board game

5. (From a few sentences A&N, V) What did Lucas need in order to play with the toys?
- Child Free Response ________
- Forced Choice Answer:
A. Instructions
*B. Someone to play with

6. (NI) How did Lucas feel about the toys at the end of the story?
- Child Free Response ________
- Forced Choice Answer:
*A. He was no longer happy with them
B. He couldn't put them down

7. (AI) Why did Lucas open the board game?
- Child Free Response ________
- Forced Choice Answer:

A. To see what was inside
*B. To play with it

Chapter 3: Baby Brother

1. (NV) Why did Lucas feel silly?
- Child Free Response ________
- Forced Choice Answer:
*A. For making a wish so quickly
B. For throwing the ball to his baby brother

2. (NI) Why did Lucas want to think harder about his next wish?
- Child Free Response ________
- Forced Choice Answer:
A. So it would come true
*B. So he wouldn't feel silly about it

3. (AV) Why was Lucas sleepy?
- Child Free Response ________
- Forced Choice Answer:
A. Because he was playing ball
*B. Because he was thinking so hard about his next wish

4. (NV) What did Lucas know when he woke up?
- Child Free Response ________
- Forced Choice Answer:
A. Where to find the bottle
*B. What to wish for

5. (NV) What was Lucas's wish?
- Child Free Response ________
- Forced Choice Answer:
A. To have more toys
*B. To have a little brother

6. (AV) What did Papa tell Lucas to do?
- Child Free Response ________
- Forced Choice Answer:
*A. To sit in the chair
B. To think harder about his next wish

7. (AV) Where did Lucas bring the baby?
- Child Free Response ________
- Forced Choice Answer:

*A. Up to his room to play
B. Over to Papa

8. (2 sentences A&N I?)Why didn't the baby catch the ball?
- Child Free Response ________
- Forced Choice Answer:
A. The baby wanted to kick the ball instead
*B. The baby was too young to play

Chapter 4: Words of Wisdom

1. (AV) Where did Papa take the boy?
- Child Free Response ________
- Forced Choice Answer:
*A. Down to the river
B. To see the baby

2. (NI) What present will make the river happy?
- Child Free Response ________
- Forced Choice Answer:
*A. No present will make it happy
B. A golden rock

3. (2 sentences A&N, V) Why did Lucas throw his lucky stone into the water?
- Child Free Response ________
- Forced Choice Answer:
*A. He didn't need his last wish to be happy
B. He used up all of the wishes

4. (AV) In this story, what must the river do to be happy?
- Child Free Response ________
- Forced Choice Answer:
*A. Share its water
B. Carry children to safety

5. (AI) What do you think will happen to Lucas when he shares?
- Child Free Response ________
- Forced Choice Answer:
*A. He will be happy
B. He will be sad

6. (AV) Which toy did Lucas pick up this time?
- Child Free Response ________
- Forced Choice Answer:

A. The ball
*B. The rattle for his baby brother

7. (NV) What did Lucas decide at the end of the story?
- Child Free Response ________
- Forced Choice Answer:
*A. To make his brother happy instead of himself
B. To throw his lucky stone into the river

8. (NV) When did Lucas become happy?
- Child Free Response ________
- Forced Choice Answer:
*A. When he heard his brother giggle
B. When his wish came true

<u>Chapter 5: Catch!</u>

1. (NV) Why was Lucas relieved when he didn't see the bottle in the beginning of the story?
- Child Free Response ________
- Forced Choice Answer:
*A. So he wouldn't make another wish
B. So he wouldn't have bad luck

2. (AV) What happened all of a sudden?
- Child Free Response ________
- Forced Choice Answer:
*A. A girl crossed Lucas's path
B. Papa appeared

3. (NV) Where does Olivia live?
- Child Free Response ________
- Forced Choice Answer:
*A. On the other side of the hill
B. On the other side of the river

4. (AV) What was Olivia holding?
- Child Free Response ________
- Forced Choice Answer:
*A. A bottle
B. A ball

5. (AI) Why did Olivia throw the bottle behind the bush?
- Child Free Response ________
- Forced Choice Answer:

A. So someone else would find it
*B. To hide it from Lucas

6. (NI) What do you think Olivia would have wished for?
- Child Free Response ________
- Forced Choice Answer:
A. Toys
*B. A friend

7. (NV) What exactly did Lucas get that he needed?
- Child Free Response ________
- Forced Choice Answer:
*A. Someone to catch the ball
B. A bottle

Best Farm (last 2 chapters)

<u>Chapter 6 – Everyone Helps</u>

1. Which animal pushed hay to the cow? AV
- Child Free Response ________
- Forced Choice Answer:
A: Cow
*B: Horse

2. At the beginning of the story, which animal wanted to eat a pumpkin? I
- Child Free Response ________
- Forced Choice Answer:
A: Horse
*B: Pig

3. What did the pig bring to the horse? AV
- Child Free Response ________
- Forced Choice Answer:
*A: Apple
B: Pumpkin

4. Who called the cat to drink milk from the bucket? NV
- Child Free Response ________
- Forced Choice Answer:
*A: Cow
B: Chicken

5. What did the chicken do after listening to the cat purr? AV

- Child Free Response ________
- Forced Choice Answer:
A: Opened the pig's pen
*B: Laid eggs

6. Where did the cat go after drinking milk? AV
- Child Free Response ________
- Forced Choice Answer:
A: To the hayloft
*B: To the chicken's nest

Chapter 7 – The Best Farm Award

1. Which animal did the judge walk to first? AV
- Child Free Response ________
- Forced Choice Answer:
A: Horse
*B: Cow

2. How many eggs were in the chicken's nest? NV
- Child Free Response ________
- Forced Choice Answer:
*A: 4
B: 6

3. Which animal was fat? NV
- Child Free Response ________
- Forced Choice Answer:
*A: Pig
B: Cow

4. Which animal ran fast? AV
- Child Free Response ________
- Forced Choice Answer:
A: Chicken
*B: Horse

5. To whom did the judge give the award? AV
- Child Free Response ________
- Forced Choice Answer:
A: Pig
*B: Farmer Manuel

6. At the end of the story, who was close to the judge? AI

- Child Free Response ________
- Forced Choice Answer:
A: Horse
*B: Farmer Manuel

Celebration (last two chapters)

<u>Chapter 5 A Gift for the Bride</u>

NV 1. What did Sofia forget?
- Child Free Response ________
- Forced Choice Answer:
*A. To buy her sister a wedding present
B. To pay for the flowers

AV 2. How many white roses did Sofia buy?
- Child Free Response ________
- Forced Choice Answer:
A. 6 (a half dozen)
*B. 12 (a dozen)

NV 3. Why did Sofia buy pink roses?
- Child Free Response ________
- Forced Choice Answer:
A. To show the love between the bride and groom
*B. To show her love for her sister, Olivia

AV 4. On Sofia's way back home the second time, what did she know?
- Child Free Response ________
- Forced Choice Answer:
*A. That she had bought everything she needed
B. That she needed to go back to the market

AV 5. Where did Sofia put the flowers after she paid for them?
- Child Free Response ________
- Forced Choice Answer:
*A. In the saddlebag
B. In the tote bag

AI 6. Why did Sofia put the flowers in the brown saddlebag?
- Child Free Response ________
- Forced Choice Answer:
*A. The other saddle bag was full of ingredients
B. She lost the tote bag

NV 7. What did Mrs. Peña think about Sofia’s gifts for her sister?
- Child Free Response ________
- Forced Choice Answer:
A. Mrs. Peña is concerned Sofia’s sister won’t like the gifts
*B. Mrs. Peña believes that Sofia’s sister will love the gifts

Chapter 6 Homecoming

AV 1. What did Sofia do before unloading the saddlebags?
- Child Free Response ________
- Forced Choice Answer:
*A. Hung the saddle and bridle on the hooks
B. Filled a vase with water

AV 2. At the end of the story, where did Sofia put the roses?
- Child Free Response ________
- Forced Choice Answer:
*A. In a vase
B. In the tote bag

AV 3. How does putting roses in a vase with water help the flowers?
- Child Free Response ________
- Forced Choice Answer:
*A. The flowers can be enjoyed longer
B. The flowers are cleaner

NV 4. What did Sofia’s mom like about the produce that Sofia had picked?
- Child Free Response ________
- Forced Choice Answer:
A. It was tasty
*B. It was fresh

AV 5. Where did Sofia’s mom examine the groceries?
- Child Free Response ________
- Forced Choice Answer:
A. At the dining table
*B. At the counter

NI 6. What will Sofia’s mom do with the ingredients?
- Child Free Response ________
- Forced Choice Answer:
A. Keep them fresh in the freezer
*B. Finish cooking dinner

AV 7. Where did Sofia bring Mancha, the horse?
- Child Free Response ________
- Forced Choice Answer:

*A. Into the corral
B. To Emilio’s house

Note: A - Action (Children manipulated images on the iPad or thought about the sentence carefully); N - Non-action (Children manipulated images on the iPad or thought about the sentence carefully); V - Verbatim (Explicitly mentioned in the story); I – Inference (Not explicitly mentioned in the story); * - The correct option on the two-alternative forced-choice.
